# Using the emotional functioning in clinical practice to detect psychological distress in patients with advanced thoracic and colorectal cancer

**DOI:** 10.1186/s12955-023-02099-w

**Published:** 2023-02-17

**Authors:** Adán Rodriguez-Gonzalez, Raquel Hernández, Patricia Cruz-Castellanos, Ana Fernández-Montes, Oscar Castillo-Trujillo, María M. Muñoz, Juana M. Cano-Cano, María J. Corral, Emilio Esteban, Paula Jiménez-Fonseca, Caterina Calderon

**Affiliations:** 1 Department of Medical Oncology, Hospital Universitario Central de Asturias, ISPA, Oviedo, Spain; 2 Department of Medical Oncology, Hospital Universitario de Canarias, Tenerife, Spain; 3 Department of Oncology Medical. Hospital, Universitario La Paz, Madrid, Spain; 4 Department of Medical Oncology, Complejo Hospitalario Universitario de Ourense – CHUO, Orense, Spain; 5 Department of Medical Oncology, Hospital General Virgen de La Luz, Cuenca, Spain; 6 Department of Medical Oncology, Hospital General Universitario de Ciudad Real, Ciudad Real, Spain; 7 Department of Clinical Psychology and Psychobiology, University of Barcelona, Barcelona, Spain

**Keywords:** Advanced cancer, Emotional function, EORTC-QLQ-C30, Psychological distress, Sensitivity, Specificity

## Abstract

**Purpose:**

Patients with advanced cancer suffer significant decline of their psychological state. A rapid and reliable evaluation of this state is essential to detect and treat it and improve quality of life. The aim was to probe the usefulness of the emotional function (EF) subscale of the European Organization for Research and Treatment of Cancer Quality of Life Questionnaire C30 (EF-EORTC-QLQ-C30) to assess psychological distress in cancer patients.

**Methods:**

This is a multicenter, prospective, observational study involving 15 Spanish hospitals. Patients diagnosed with unresectable advanced thoracic or colorectal cancer were included. Participants completed the Brief Symptom Inventory 18 (BSI-18), the current the gold standard, and the EF-EORTC-QLQ-C30 to assess their psychological distress prior to initiating systemic antineoplastic treatment. Accuracy, sensitivity, positive predictive value (PPV), specificity, and negative predictive value (NPV) were calculated.

**Results:**

The sample comprised 639 patients: 283 with advanced thoracic cancer and 356 with advanced colorectal cancer. According to the BSI scale, 74% and 66% displayed psychological distress with an EF-EORTC-QLQ-C30 accuracy of 79% and 76% in detecting psychological distress in individuals with advanced thoracic and colorectal cancer, respectively. Sensitivity was 79 and 75% and specificity was 79 and 77% with a PPV of 92 and 86% and a NPV of 56 and 61% (scale cut-off point, 75) for patients with advanced thoracic and colorectal cancer, respectively. The mean AUC for thoracic cancer was 0.84 and, for colorectal cancer, it was 0.85.

**Conclusion:**

This study reveals that the EF-EORTC-QLQ-C30 subscale is a simple and effective tool for detecting psychological distress in people with advanced cancer.

## Introduction

In recent years, improvements in cancer treatments and early detection have led to better prognosis and survival for subjects with thoracic and colorectal cancer [1–4]. However, a significant number of them still develop advanced, untreatable cancers and undergo treatments to extend survival, but often at the expense of diminished quality of life due to treatment toxicity and dosing [5–7]. This decline in quality of life is especially pronounced in advanced and end-of-life patients [6–8].

Cancer patients suffer a high incidence of psychological distress, with rates ranging from 42 to 90% depending on the type of cancer, stage, and population studied [9–11]. According to Carrozzino [12], psychological distress can be defined as a subjective, multi-dimensional, transdiagnostic construct that encompasses feelings of discomfort, demoralization, mental pain, anguish, somatic symptoms, and self-criticism. These individuals also exhibit a high prevalence of depression, anxiety, and adjustment disorders [13–15]. This is especially true in patients with thoracic and colorectal cancer, which are two of the leading types of cancer in terms of incidence and mortality [6, 8, 16]. Emotional distress has shown strong associations with decreased physical activity and symptoms such as pain and fatigue in patients with lung [17, 18], gastric [6], and colorectal cancer [19]. Psychological state assessment is common in clinical trials [20] as one of the domains included in quality of life questionnaires [21].

This psychological distress can affect treatment, worsening its tolerability, potentially impacting outcomes, increasing the risk of suicide, and early patient demise [22]. Such is the negative impact of these psychological symptoms on activities of daily living and health-related quality of life (HRQoL) in cancer patients that the American Society of Clinical Oncology (ASCO) recommends implementing quick screening instruments that assess psychological distress in cancer patients [23] and measures to mitigate its impact.

For the oncologist, the importance of having instruments available to rapidly appraise the person’s psychological state lies in the fact that they typically have limited time to care for the patient during clinical visits and have to assess many symptoms and complications associated with cancer and its treatment, oftentimes making it difficult to adopt a comprehensive and effective approach to the psychological sphere. On the other hand, because of their general physical and psychological state, individuals with advanced cancer have a more limited ability to concentrate and answer questionnaires than the general population. Therefore, the emotional function (EF) subscale of the European Organization for Research and Treatment of Cancer Quality of Life Questionnaire C30 (EORTC-QLQ-C30) can serve as a rapid screening scale to assess psychological distress in subjects with advanced cancer [24], especially since the EORTC-QLQ-C30 is the most used scale to measure quality of life in clinical trials in Spain [24, 25] and in the rest of the world [26, 27].

When using the EF subscale of the EORTC-QLQ30 (EF-EORTC-QLQ30) to identify people with psychological distress, a cut-off point < 66.7 is often used, following the distribution of scores in the general population [21]. Nevertheless, some authors have found a lower mean score in cancer patients [28], suggesting a cut-off point of < 0.46 [29], while others recommend using a cut-off point < 0.75 [28] or even < 90 [30] to identify cancer patients with psychological distress. There is currently no consensus on the standard cut-off point in subjects with cancer and there are no studies that have established a cut-off point in the scenario of advanced cancer. This study sought to determine the specificity, the sensitivity, and cutoff of the Emotional functioning subscale of the EORTC QLQ-C30 to detect psychological distress in patients with advanced thoracic cancer and colorectal cancer. The study assesses the hypothesis that a two-item component of the emotional functioning scale can be useful as an initial screening measure to identify advanced cancer patients at risk for psychological distress.

## Methods

### Study design and patients

NEOetic is an observational, prospective, multi-institutional study involving 15 medical oncology departments in Spain and promoted by the Bioethics Group of the Spanish Society of Medical Oncology (SEOM). The study protocol complied with the provisions of the Declaration of Helsinki, was approved by the ethics committees of each hospital and by the Spanish Agency of Medicines and Health Products (AEMPS; identification code: ES14042015). All eligible patients were identified by oncologists. Participants were informed that their participation was voluntary, anonymous, and confidential. Written informed consent was obtained from all participants prior to data collection. Participants were aged 18 years or older, had histologically confirmed, unresectable advanced thoracic or colorectal cancer, and were candidates for systemic therapy. Individuals with severe mental illness that could compromise study adherence were excluded. Thoracic cancer included all cancers occurring in the thoracic cavity, including cancers of the lung, pleura (mesothelioma), thymus, and trachea. Colorectal cancer included all cancers occurring in the large intestine from the ileocecal valve to the lower rectum. All cancers included were of epithelial origin; neoplasms of other types, such as neuroendocrine tumors, hematological tumors, and sarcomas were therefore excluded as their management and prognosis are different from those of carcinomas.

### Measures

The data collection procedures were similar in all hospitals. Clinical variables (type of tumor, pathological and molecular variables, cancer stage, treatment, performance status, and comorbidities) were obtained from the medical records and collected and updated by the medical oncologist who informed the patient of their diagnosis and prescribed antineoplastic therapy. These variables were compiled through a web platform (www.neoetic.es).

The subjects provided information concerning their age, sex, level of education, and occupational status, as well as the EORTC-QLQ-C30 an BSI-18 scales. The oncologist gave the questionnaires to each subject after shared treatment decision making. Questionnaires were filled out at home and handed in to the study assistants at the next appointment before starting systemic antineoplastic treatment.

The EF-EORTC-QLQ30 consists of four items that probe affective aspects of anxiety, depression, and general distress based on patients’ perceptions of feeling tense, worried, depressed, and irritable [21]. Items are scored on a four-point Linkert scale from 0 (“not at all”) to 4 (“very much”) over a one-week period. Raw scores are transformed into a scale from 0 to 100 with higher scores indicating better functioning. For the purpose of this work, we established that a score ≤ 75 indicated a psychological problem and > 75 meant “no problem” [31, 32]. In our sample, 49% had an EF score > 75; Cronbach’s α for the scale was 0.89. The Spanish version of the EF-EORTC-QLQ-C30 has demonstrated satisfactory reliability and validity in the Spanish population and a completion time of less than 3 min [24].

The BSI-18 is one of the most widely used instruments to assess psychological distress [33]. It is an 18-item scale containing three groups of six questions each that comprise the anxiety, depression, and somatization subscales [33]. It is scored on a 5-point Likert scale (0–4) based on a one-week recall period. The overall Global Severity Index (GSI) score ranges from 0–72 with higher scores evidencing greater anxiety or depression. Raw scores are converted to T-scores based on sex-specific normative data. In the present study, Cronbach’s α values for the anxiety and depression scales were 0.87 and 0.74, respectively. The Spanish version of the BSI-18 has demonstrated its reliability and validity in Spanish patients [34].

### Statistics

The BSI-18 questionnaire was used as the “gold standard” for comparison with the EF-EORTC-QLQ-C30. The BSI-18 applies the clinical case rule (39) originally developed for the SCL-90 to identify individuals with significant psychological distress (T-cut-off ≥ 63) [33]. According to our gold standard test, psychological distress designated by the EF-EORTC-QLQ-C30 was defined as true positive (TP, correctly identified as case), true negative (TN, correctly identified as non-case), false positive (FP, incorrectly identified as case), and false negative (FN, incorrectly identified as non-case). The following measures were calculated: (1) the number of correctly identified patients with psychological distress (overall test accuracy [TP + TN]/[TP + TN + FP + FN]); (2) the proportion of correctly identified positives (true positive/sensitivity rate, TP/[TP + FN]); (3) the proportion of correctly identified negatives (true negative/specificity rate, TN/[TN + FP]); (4) the proportions of TP (BSI-18) results (EF-ORTC-QLQ-C30) (positive predictive value, TP/[TP + FP]), and (5) the proportions of TN results (negative predictive value, TN/[TN + FN]). The discriminatory ability of the EF-EORTC-QLQ30 score was calculated using the area under the receiver operating characteristic (ROC) curve (AUC). The AUC summarized the ability of the EF-EORTC-QLQ-C30 to discriminate between patients with and without psychological distress. A higher AUC indicated better discriminatory capacity. We used a threshold AUC ≥ 0.70 for the EF-EORTC-QLQ-C30, which was also the standard used for our previous analysis [30, 35]. Analyses were performed with the IBM-SPSS 23.0 statistical software package for Windows PC.

## Results

### Patient baseline characteristics

A total of 660 consecutive patients agreed to participate in the study between February 2020 and December 2022. Twenty-one patients were excluded as they failed to meet the inclusion criteria. This resulted in a final sample of 639 participants of whom 283 had unresectable advanced thoracic cancer and 356 had unresectable advanced colorectal cancer.

Demographic and clinical characteristics are exhibited in **Table ****1**. The advanced thoracic cancer cohort included cancer of the lung (82%, n = 232), esophagus (15%, n = 42), and pleura (3%, n = 9). The advanced colorectal cancer group included colon (80%, n = 284), rectal (18%, n = 64), and intestinal cancer (2%, n = 8). The subjects with a thoracic cancer were predominantly male (62%) with a mean age of 65.6 years (standard deviation (SD) = 9.5) and two thirds had stage IV cancer (78%). Those with colorectal cancer were also mainly male (61%) with a mean age of 66.0 years (SD = 10.6) and most had stage IV cancer (85%). No significant differences in psychological distress were revealed regarding age, gender, marital status, and education in individuals with thoracic or colorectal cancer.

**Table 1:** Patient baseline demographic characteristics

Demographic characteristics	Unresectable advanced thoracic cancer (*n* = 283)	Unresectable advanced Colorectal Cancer (*n* = 356)
Age (Mean ± Standard Deviation)	65.6 ± 9.5	66.0 ± 10.6
Gender (n, %)		
Male	176 (62)	218 (61)
Female	107 (38)	154 (39)
Marital status		
Married or partnered	219 (85)	281 (86)
Not partnered	64 (15)	75 (14)
Education		
≤ Primary	112 (39)	185 (52)
> High School	171 (61)	171 (48)
Employed		
Yes	147 (52)	199 (56%)
No (retired or unemployed)	136 (48)	157 (44%)
Clinical characteristics		
Stage (n, %)		
Locally advanced	63 (22)	54 (15)
IV	220 (78)	302 (85)
Histology		
Adenocarcinoma	146 (52)	300 (84)
Others	137 (48)	56 (16)
Estimated survival		
Less than 12 months	70 (25)	101 (28)
More than 12.1 months	213 (745)	255 (78)
First diagnosis of cancer		
No (recurrence)	37 (13)	59 (16)
Yes	246 (87)	297 (84)
Systemic treatment (n, %)		
Chemotherapy	197 (70)	337 (95)
Others without chemotherapy	86 (30)	19 (5)

### Screening for psychological distress

**Fig. 1:**
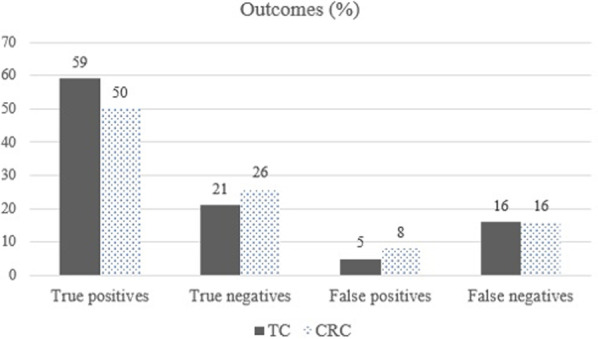
Outcomes: true positive (TP), true negative (TN), false positive (FP), and false negative (FN) for unresectable advanced thoracic (TC) and colorectal cancer (CRC)

In total, 210 patients (74%) with advanced thoracic cancer and 236 (66%) with colorectal cancer showed psychological distress according to the BSI-18 scale which, as previously mentioned, was deemed the gold standard. The mean BSI-18 score was 67.1 (SD = 7.5) in thoracic cancer and 66.1 (SD = 7.1) among participants with advanced colorectal cancer. The accuracy of the EF-EORTC-QLQ-C30 for detecting psychological distress was 79% among the thoracic cancer group and 76% in the colorectal cancer group using a cut-off point < 75. FPs were detected in 15 subjects with thoracic cancer and 27 with colorectal cancer. Considering these FPs, specificity was 79% in thoracic cancer and 77% in colorectal cancer. FNs were detected in 44 subjects with thoracic cancer and in 58 with colorectal cancer (Fig. 1). As a result of these FNs, sensitivity was 79% in thoracic cancer and 75% in colorectal cancer.

Moreover, the positive predictive value was 92% for thoracic cancer and 87% for colorectal cancer, while the negative predictive value was 56% for thoracic cancer and 61% for colorectal cancer. The AUC for thoracic cancer was 0.84 (95% confidence interval (CI), 0.78–0.89) and 0.85 (95% CI, 0.80–0.89) for colorectal cancer (Table 2).

**Table 2:** Detecting psychological distress with the Emotional Function subscale of the European Organization for Research and Treatment of Cancer Quality of Life Questionnaire Core-30 (EF-EORTC-QLQ-C30)

	Unresectable advanced thoracic cancer (n = 283)	Unresectable advanced colorectal cancer (n = 356)
Psychometric properties		
Accuracy	0.791	0.761
Sensitivity	0.790	0.754
Specificity	0.794	0.775
Positive predictive value	0.917	0.868
Negative predictive value	0.568	0.619
Area Under the Curve (AUC)*	0.78–0.89	0.80–0.89

*Relationship between EF-EORTC-QLQ-C30 and Brief Symptom Inventory 18 (BSI-18)

Using a cut-off point < 90 for the EF-EORTC-QLQ-C30 as suggested by Snyder et al. [30], the positive predictive value decreases in both the thoracic cancer and the colorectal cancer group (89% and 83%, respectively), while the negative predictive value increases in both (67% and 71%, respectively). Therefore, the use of a cut-off point < 75 appears to perform better than < 90.

## Discussion

This study demonstrates that the EF-EORTC-QLQ-C30 subscale is a simple and effective tool to detect psychological distress in patients with advanced cancer showing an accuracy of 79% and 76% for subjects with thoracic and colorectal cancer, respectively.

These results confirm not only the utility of this tool but also the high incidence of psychological distress in this population and therefore the need for routine assessment to better diagnose and care for these individuals [9–11]. In fact, HRQoL assessment has been common practice in many clinical trials for years, and emotional functioning is a domain included in most HRQoL measures [29]. Currently, several international guidelines recommend the use of brief screening measures to detect and manage psychological distress in cancer patients [20, 36, 37]. While there are validated scales to measure this parameter, most of them are complex and difficult to apply, and a quick and simple scale such as EF-EORTC-QLQ-C30 is needed to do so more routinely.

This screening aids in the early detection of psychological distress so that interventions can be implemented sooner and repercussions during the disease can be avoided [8, 15]. The relevance of this derives from the fact that clinically significant levels of depressive symptoms have been associated with poorer survival in cancer patients [38, 39]. Thus, Siwik et al. [38] found that the presence of relevant depressive symptoms correlated with worse survival in lung cancer patients. In addition, emotional distress in this population may entail a poorer prognosis [40] given that, as distress increases, coping deteriorates, adherence to treatment worsens [41, 42], and the risk of disease progression or recurrence increases [43, 44].

This assessment pre- and post-cancer treatment, as recommended by ASCO [45], would enable different profiles of patients with psychological distress to be established according to age, gender, and other characteristics. In two studies of patients receiving antineoplastic treatment, younger individuals (40–55 years) reported more anxiety and depression than patients older than 70 years [45, 46] and women reported more of these symptoms than men [46]. Andersen et al. [47] studied the trajectories of anxiety and/or depression symptoms in patients with stage IV non-small cell lung cancer. Anxious and depressive symptoms decreased significantly over time following diagnosis, and persistence of depression was associated with shorter survival. These studies suggest that psychological distress can be detected and assessed, that it may be reversible, doing so, can hold immediate benefits for emotional well-being on survival in the mid-term,. Therefore, it is worthwhile for the oncologist to have a short and rapid screening tool to evaluate the emotional state of cancer patients and ensure proper interpretation of the scores [35, 37, 48].

The present study reveals how the EF-EORTC-QLQ-C30 (cut-off point < 75) is practical to detect psychological distress quickly in patients with advanced thoracic and colorectal cancer. In our sample, this scale has a sensitivity of 79% and 75% and a specificity of 79% and 77% for thoracic and colorectal cancer, respectively, considering the BSI-18 as the gold standard of measurement.

Generally, a cut-off point < 66.78 on the EF-EORTC-QLQ-C30 is used to identify cancer patients with psychological problems in line with the distribution of scores in the general population [22]. Snyder et al. [30] recommend using a score < 90. However, we prefer to apply a score < 75, as scores between 66 and 75 would leave some 30% of individuals misidentified. Giesinger et al. [28] have defined a threshold of 70 points on this scale. Nevertheless, the use of this cut-off point in our sample of cases with unresectable advanced cancer appears to perform worse than the one we propose; i.e., 75. These differences in the choice of cut-off point might be related to different characteristics of the populations analyzed in the studies. Therefore, we believe that, in future studies, it would be compelling to probe the influence of clinical and sociodemographic variables in establishing the cut-off point for this subscale. Similarly, it is important that future studies use clinimetric criteria to ensure that measures are accurate, valid, sensitive to change, and useful to assess patients’ experiences and tracking their progress over time [49].

The strengths of this study are its large sample size (639 patients), the representativeness of the sample (cases from 15 hospitals throughout Spain), and the fact that the incidence of emotional distress was found in a specific population of patients with unresectable (incurable) advanced cancer at a specific time, following diagnosis and prior to initiating systemic treatment. The incidence was 71–75% in patients with unresectable advanced thoracic and colorectal cancer, similar to figures reported in other series, thereby highlighting the relevance of this issue [48, 50]. This study also has limitations that should be considered. First, its cross-sectional nature. The evaluation was performed before starting antineoplastic treatment; consequently, there may be patients who emotionally adapt to this situation during treatment and others who, on the contrary, get worse. Therefore, for the future, it would be important to assess whether the EF-EORTC-QLQ-C30 scale is also useful to detect emotional distress during and after cancer treatment. Second, the psychometric properties and sensitivity of the EF-EORTC-QLQ-C30 were adequate to detect psychological distress in cancer patients; consequently, the cut-off point used in this research should be validated in patients with other types of cancer and at other stages. Third, the reference test was another questionnaire, the BSI-18, and no psychiatric assessment or clinical diagnosis was made.

In conclusion, the EF-EORTC-QLQ-C30 was 79% and 76% accurate in detecting psychological distress in patients with advanced thoracic and colorectal cancer, respectively. Therefore, this short, useful scale, with an accurate cut-off point, can help healthcare professionals identify individuals with emotional problems requiring specialized care. The brevity of this scale makes it ideal for longitudinal administration, comparison of results from different studies and analysis of the impact of different treatments and interventions.

## Data Availability

The datasets generated and analyzed during the current study are not publicly available for reasons of privacy. They are, however, available (fully anonymized) from the corresponding author on reasonable request.
